# miR-410-3p Suppresses Cytokine Release from Fibroblast-Like Synoviocytes by Regulating NF-κB Signaling in Rheumatoid Arthritis

**DOI:** 10.1007/s10753-018-0896-2

**Published:** 2018-09-21

**Authors:** YueJiao Wang, NeiLi Xu, Shuai Zhao, Ting Jiao, WenYi Fu, LiLi Yang, Ning Zhang

**Affiliations:** The Department of Rheumatology, Shengjing Hospital of China Medical University, Shenyang, Liaoning China

**Keywords:** rheumatoid arthritis, miR-410-3p, fibroblast-like synoviocytes, cytokines, NF-κB signaling

## Abstract

miR-410-3p acts as an oncogene or a tumor suppressor in some malignancies. However, its role in rheumatoid arthritis (RA) is unknown. The study was conducted to investigate the effect of miR-410-3p on the pathogenesis of RA. Real-time RT-PCR was used to determine the mRNA levels of miR-410-3p in synovial tissues and fibroblast-like synoviocytes (FLSs). An ELISA was performed to examine the production levels of tumor necrosis factor (TNF)-α, interleukin (IL)-1β, IL-6, and matrix metalloproteinase (MMP)-9. Western blotting was conducted to determine the protein levels of IκB-α, p-IκBα, p65, and p-p65. Nuclear factor (NF)-κB activation and nuclear translocation assays were performed to confirm the activation of NF-κB. We found that the expression level of miR-410-3p was downregulated in synovial tissues and FLSs from RA. Overexpression of miR-410-3p significantly reduced the secretion of TNF-α, IL-1β, IL-6, and MMP-9 in human RA fibroblast-like synoviocytes (HFLS-RA); whereas miR-410-3p inhibition increased the expression levels of these cytokines. Furthermore, miR-410-3p suppresses the activation of NF-κB signaling pathway. Moreover, NF-κB inhibitor restored the elevation of TNF-α, IL-1β, IL-6, and MMP-9 induced by miR-410-3p inhibition. Our results demonstrate that miR-410-3p acts an inflammatory suppressor in the pathogenesis of RA by regulating the NF-κB signaling pathway. These data suggest a novel function of miR-410-3p and provide insight into the complex mechanisms involved in RA.

## INTRODUCTION

Rheumatoid arthritis (RA) is a chronic autoimmune inflammatory disease that causes progressive articular damage, functional loss, and comorbidity. RA affects approximately 1% of the population, can develop at any age, and is more prevalent in women than in men [1]. An improved understanding of RA pathogenesis has led to several therapeutic options; however, additional studies are needed to avoid side effects and improve the quality of life of RA patients [2]. Current treatment strategies involve traditional disease-modifying anti-rheumatic drugs and novel biologic agents targeting T cells, B cells, pro-inflammatory cytokines including tumor necrosis factor (TNF) or interleukin (IL)-1, and tyrosine kinase activity both markedly improve the clinical outcomes of RA patients [2, 3]. However, at least 30% of RA patients show poor responses to the available therapies, suggesting that novel mediators are needed for targeting of other disease-specific pathways or cell lineages.

Fibroblast-like synoviocytes (FLSs), also known as synovial fibroblasts, are a quite unique cell type that distinguishes RA from other inflammatory conditions of the joints and are key effector cells in the pathogenesis of RA [4]. FLSs from RA patients exhibit a unique progressive phenotype that increases invasiveness into the extracellular matrix and further exacerbates joint damage. This invasiveness of FLSs is considered equivalent to that of tumor cells [5]. Numerous studies showed that FLSs participate in cell inflammation by secreting cytokines and chemokines, particularly TNF-α, IL-1β, and IL-6, and result in joint tissue damage in RA [6]. Furthermore, inflammatory cytokines secreted by FLSs increase the total number of FLSs and promote the development of systemic inflammation, such as chronic anemia and cardiovascular disease [7]. Therefore, understanding the molecular mechanism of dysregulation of cytokines expression in RA patients, particularly in FLSs, may provide novel treatment strategies.

MicroRNAs (miRNAs) are endogenous small noncoding RNA molecules that modulate the expression of multiple protein-encoding genes at the post-transcriptional level [8]. Accumulating evidence has shown that these small RNA molecules play important roles in various pathological conditions, including rheumatic and other autoimmune diseases [9, 10]. Several studies suggested that miRNAs are involved in controlling the inflammatory response of immune and non-immune cells. For instance, miR-124 mediates the anti-inflammatory action by suppressing production of the pro-inflammatory cytokines IL-6 and TNF-α [11]. Lucas Philippe et al. observed dysregulated expression of miR-20a decreased IL-6 and CXCL10 released by RA FLSs and IL-1β and TNF-α by activated THP-1 cells [12]. Recent studies suggested that miR-410-3p is involved in inflammation, angiogenesis, and tumorigenesis [13–15]. Furthermore, as a key regulatory factor in the pathogenesis of systemic lupus erythematosus (SLE), miR-410-3p was found to be decreased in T cells of SLE patients and regulated the expression of IL-10 [16]. However, no studies have examined miR-410-3p expression and its roles in RA.

In this study, we explored the expression of miR-410-3p in FLSs and synovial tissues of RA patients, its role in inflammatory cytokine secretion in RA-FLSs, and the underlying mechanisms.

## METHODS

### Cell Lines and Tissues

Human RA fibroblast-like synoviocytes (HFLS-RA) and human normal fibroblast-like synoviocytes (HFLS) were purchased from Jennio Biotech Co., Ltd. (Guangzhou, China). HFLS-RA were cultured in DMEM (Gibco, Life Sciences, MD), and HFLS were cultured in MEM (Gibco), supplemented with 10% heat-inactivated fetal bovine serum (FBS) (Gibco), penicillin (final concentration, 100 U/ml), and streptomycin (final concentration, 0.1 mg/ml) (all from Hyclone, Logan, UT), in a humidified atmosphere of 5% CO_2_ and 95% air at 37 °C. Cells were passaged every 4–5 days. Cells from the 3rd–8th passages were used for the following experimental procedures.

Synovial tissues were obtained from RA patients at the time of arthroscopic biopsy or total joint replacement from the Department of Orthopaedics in the Shengjing Hospital of China Medical University. All five RA patients met the 1987 American College of Rheumatology (ACR) criteria for the classification of RA [17]. Four control synovial tissue samples were obtained from patients with joint trauma during routine arthroscopy or open joint surgery for diagnostic and therapeutic procedures. All patients were informed of the purpose of the study and gave written consent. The study was approved by the Ethics Committee of the Shengjing Hospital of China Medical University.

### Transfection Experiments

The experimental setup consisted of five groups: the blank group, miR-410-3p mimics group (transfected with miR-410-3p mimics), mimics-negative control (transfected with miR-410-3p mimics-negative control sequence), miR-410-3p inhibitor group (transfected with miR-410-3p inhibitor), and inhibitor-negative control (transfected with miR-410-3p inhibitor-negative control sequence). miR-410-3p-related sequences were synthesized by GenePharma (Shanghai, China). HFLS-RA cells were seeded into six-well plate, transfected using Lipofectamine™ 3000 Reagent (Invitrogen, USA). The respective miR-410-3p sequences and Lipofectamine 3000 were mixed separately with Opti-MEM (Invitrogen) and then incubated together at room temperature for 10 min under serum-free conditions to form transfection complexes. The HFLS-RA was washed twice with PBS and the corresponding transfection complexes were added to each well. Complete DMEM media was replaced 6 h later. The transfection efficiency was verified by fluorescence microscopy at 24 h, and cells at 48 h and 72 h post-transfection were used for further analysis.

### miR-410-3p Quantification

HFLS-RA cells were first transfected with miR-410-3p mimics, inhibitor, or NC for 48 h. Quantification of miR-410-3p was performed with real-time RT-PCR as previously described with modifications [18]. In brief, total RNA containing small-size RNA was isolated or extracted from synovial tissues and cells with RNAiso Plus reagent (Takara, Japan). Total RNA (3 μg) was reversely transcribed to cDNA using a Mir-XTM miRNA First-Strand Synthesis kit (Takara). Next, the cDNA was amplified with a SYBR® Premix Ex Taq™ II (Tli RNaseH Plus) kit (Takara). U6 was used as an internal control and the relative expression of miR-410-3p was calculated using the 2-ΔΔ*CT* method. The PCR primers were commercially obtained from Sangon Biotech (Shanghai, China).

### Western Blot

HFLS-RA cells were first transfected with miR-410-3p mimics, inhibitor, or NC for 72 h, and then were harvested and lysed using RIPA buffer (Beyotime Biotechnology, Shanghai, China). The protein concentration was determined with a BCA kit (Beyotime Biotechnology) according to the protocol. Equal amounts of protein corresponding to approximately 30 μg were subjected to SDS-PAGE. Then, proteins were transferred to polyvinylidene difluoride (PVDF) membranes and incubated with 5% BSA for 2 h. Diluted primary antibodies (IκB-α, p-IκBα, p65, and p-p65; Cell Signaling Technology, USA; GAPDH and β-actin; ZSGB-BIO, China) were added into the membranes, and the mixture was incubated at 4 °C overnight. The membranes were washed with Tris-buffered saline Tween-20 (TBST). Horseradish peroxidase-labeled secondary antibodies (ZSGB-BIO) were added and the mixture was incubated at room temperature for 2 h. The membrane was then washed with TBST three times for 10 min once. The bands were visualized by enhanced chemiluminescence (ECL) according to the instructions (Millipore). The protein bands were quantitatively analyzed by using Image J software, normalized to GAPDH or β-actin.

### Assessment of NF-κB Nuclear Translocation

NF-κB activation and nuclear translocation assay was performed according to the reagent manufacturer’s instructions (Beyotime Biotechnology). Briefly, 24 h after being plated in six-well plate, HFLS-RA cells were transfected with miR-410-3p mimics, inhibitor, or NC for 72 h, then fixed and blocked at room temperature. After incubation with rabbit anti-p65 NF-κB antibody overnight at 4 °C, cells were added with fluorescent secondary antibody. Eventually, the nuclear was dyed with DAPI (Beyotime Biotechnology). The fluorescence microscope was used to take pictures.

### Enzyme-Linked Immunosorbent Assay

HFLS-RA cells were seeded into 24 well plates and transfected with miR-410-3p mimics, inhibitor, or NC for 48 h, with or without BAY 11-7082 treatment (Beyotime Biotechnology). The concentrations of TNF-α, IL-1β, IL-6, and MMP-9 in the cell culture supernatants were determined by a sandwich enzyme-linked immunosorbent assay (ELISA) (R&D Systems, Minneapolis, MN) according to the manufacturer’s instructions. A standard curve was performed for each plate and used to calculate the absolute concentrations of the indicated cytokines.

### Statistical Analysis

All data were expressed as the mean ± standard deviation from at least three independent experiments and normally distributed. The differences between two groups were analyzed using the two-tailed Student *t* test. For the differences among three groups, one-way ANOVA followed by Tukey’s multiple comparisons test was used. *P* values below 0.05 was considered as statistically significant (ns, non-significant, *p < 0.05). All calculations were performed using the GraphPad Prism version 7.00 (GraphPad Software, Inc., San Diego, CA, USA).

## RESULTS

### miR-410-3p Is Reduced in Synovial Tissues and Fibroblast-Like Synoviocytes in RA Patients

**Fig. 1:**
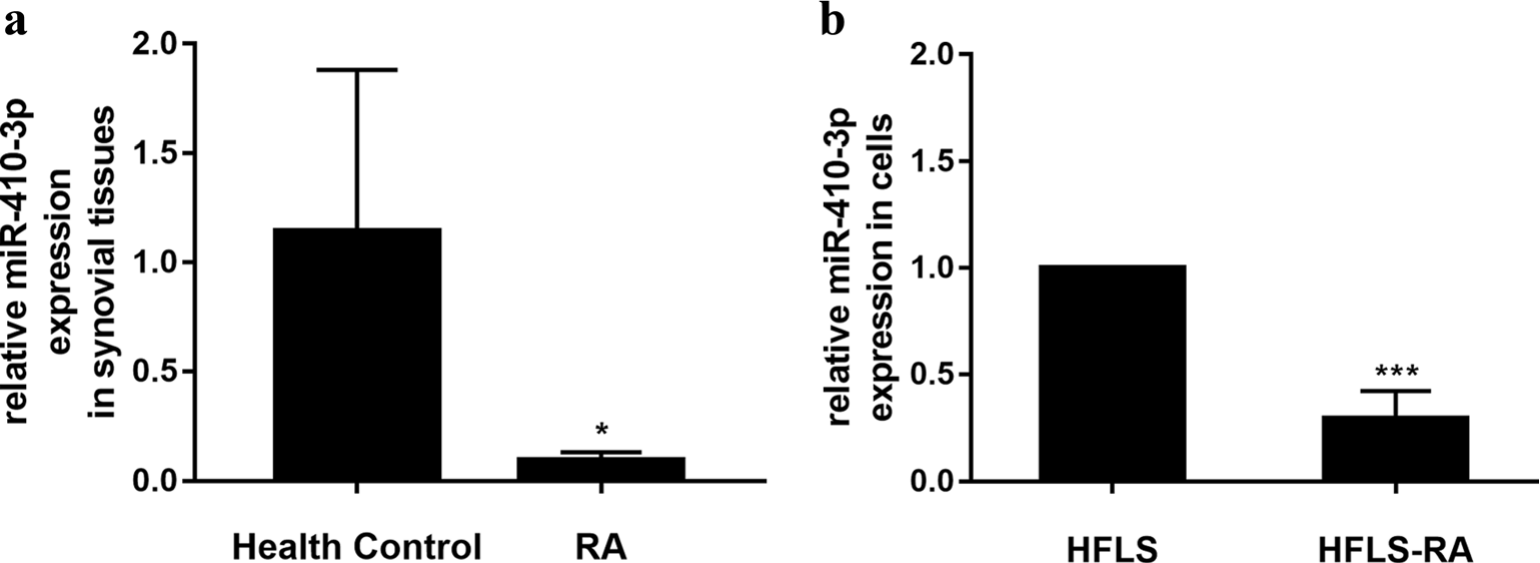
miR-410-3p is reduced in synovial tissues and FLSs in RA patients. Synovial tissue samples were isolated from non-RA controls and RA patients, and miR-410-3p level was determined by real-time RT-PCR. All samples were performed in triplicates for each condition. Data shown were mean ± SD of three independent experiments. **a** Expression of miR-410-3p in synovial tissues of 4 HCs and in the 5 patients with RA. **b** Expression of miR-410-3p in HFLS and HFLS-RA.*p < 0.05, ***P < 0.001, compared with control.

We first tested the miR-410-3p expression in synovial tissues in RA patients. Synovial tissue samples were isolated from both non-RA controls and RA patients, and miR-410-3p expression was quantified. As shown in Fig. 1, the miR-410-3p expression was significantly decreased in the synovial tissues from RA patients (*p* < 0.05). Next, we compared the miR-410-3p level between two synovial cell lines, and the miR-410-3p level was lower in HFLS-RA (*p* < 0.001), indicating that aberrant miR-410-3p expression is associated with the pathogenesis of RA.

### Effect of miR-410-3p Expression on HFLS-RA Cytokine Release

**Fig. 2:**
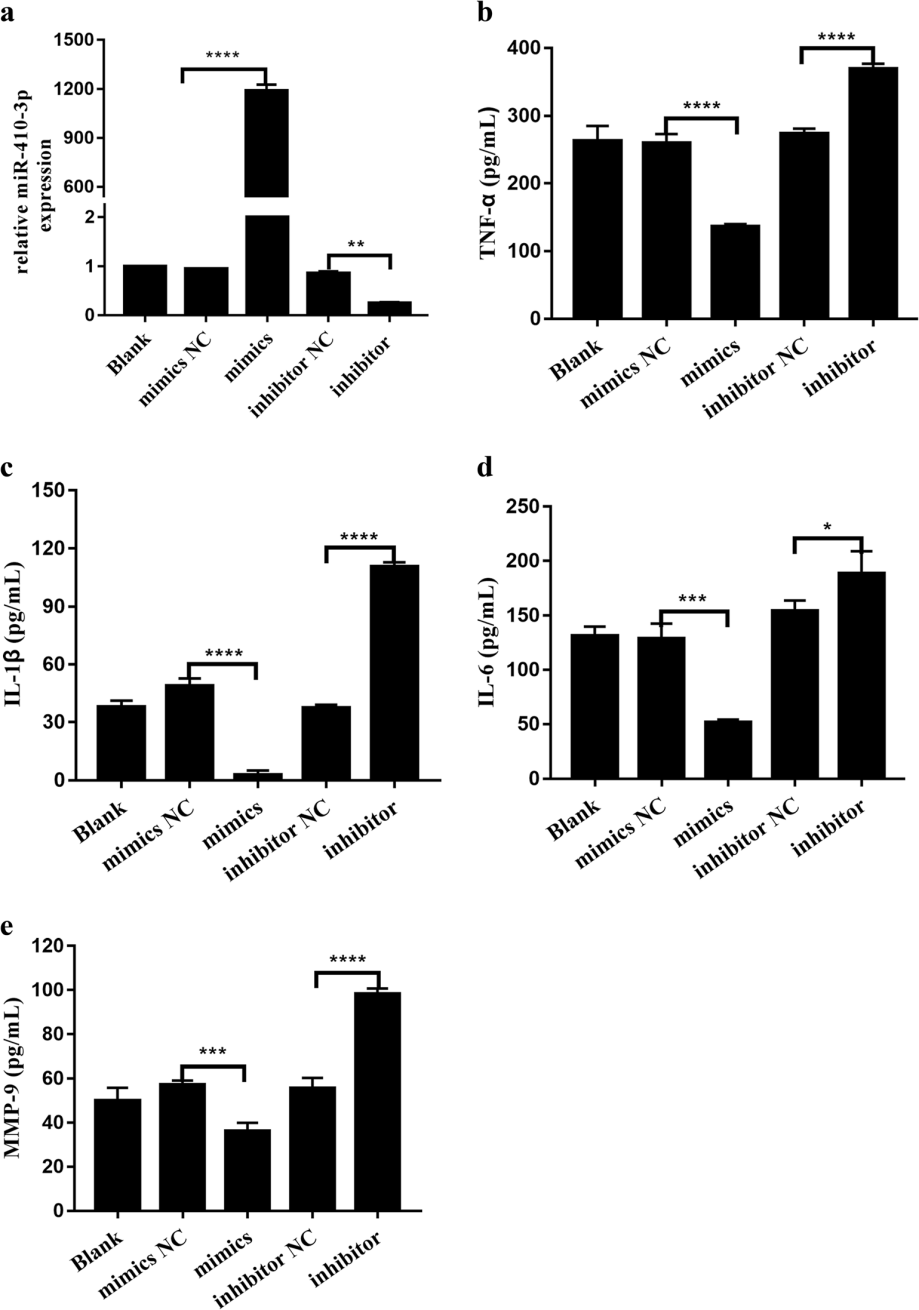
Effect of miR-410-3p expression on HFLS-RA cytokine release. miR-410-3p mimics and inhibitor were designed and transfected into HFLS-RA followed by measuring the expression of miR-410-3p by real-time RT-PCR and the secretion of TNF-α, IL-1β, IL-6, and MMP-9 by ELISA. **a** Expression levels of miR-410-3p in HFLS-RA after transfection. **b**–**e** Relative expression levels of TNF-α, IL-1β, IL-6, and MMP-9 in HFLS-RA after transfection. *p < 0.05, **p < 0.01, ***p < 0.001, ****p < 0.0001, compared with control.

To measure the effect of miR-410-3p levels, miR-410-3p mimics and inhibitor were used to manipulate the expression level of miR-410-3p in HFLS-RA. Total RNA was extracted at 48 h after transfection to detect the relative expression of miR-410-3p. As shown in Fig. 2a, miR-410-3p level was reduced to approximately 20% after miR-410-3p inhibitor transfection (*p* < 0.01), whereas it was markedly increased after miR-410-3p mimics transfection (*p* < 0.0001).

At 48 h after transfection, cytokines levels, including those of TNF-α, IL-1β, IL-6, and MMP-9, secreted from HFLS-RA were evaluated by ELISA. It was presented that TNF-α, IL-1β, IL-6, and MMP-9 levels were clearly downregulated in HFLS-RA transfected with miR-410-3p mimics (*p* < 0.05), whereas they were significantly elevated in HFLS-RA transfected with miR-410-3p inhibitor (*p* < 0.05) (Fig. 2b–e). These results indicate that miR-410-3p suppressed the secretion of inflammatory cytokines in HFLS-RA.

### Effect of miR-410-3p in HFLS-RA on the NF-κB Signaling Pathway

**Fig. 3:**
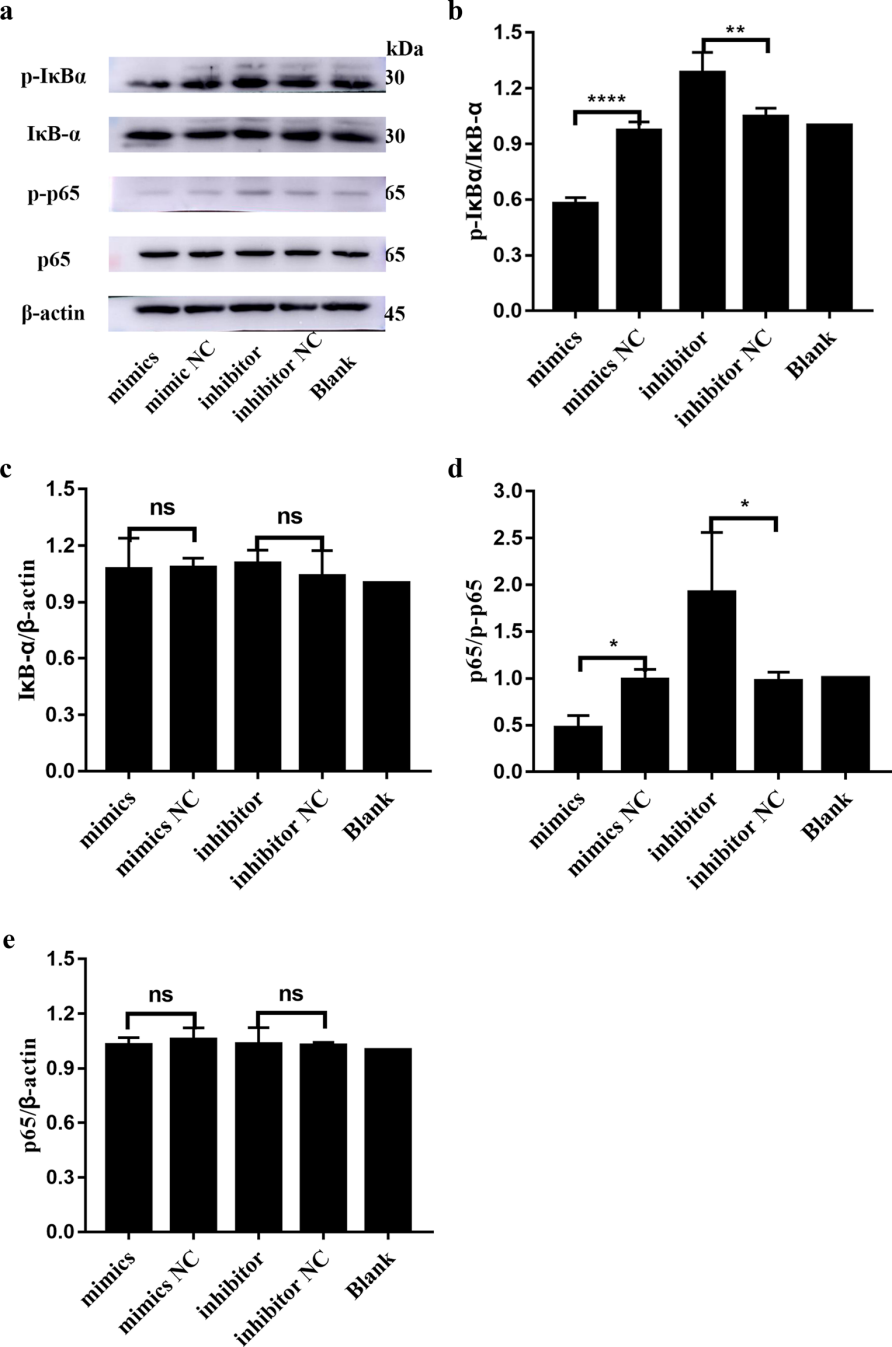
Effect of miR-410-3p in HFLS-RA on NF-κB signaling pathway. miR-410-3p mimics and inhibitor were transfected into HFLS-RA followed by measuring the expression of p-IκBα, IκB-α, p-p65, and p65 levels by western blot. **a** Western blot detection of p-IκBα, IκB-α, p-p65, and p65 at 72 h after miR-410-3p transfection. **b**–**e** Histograms showing the expression of p-IκBα, IκB-α, p-p65, and p65 levels detected by western blot. *p < 0.05, **p < 0.01, ****p < 0.0001, compared with control.

**Fig. 4:**
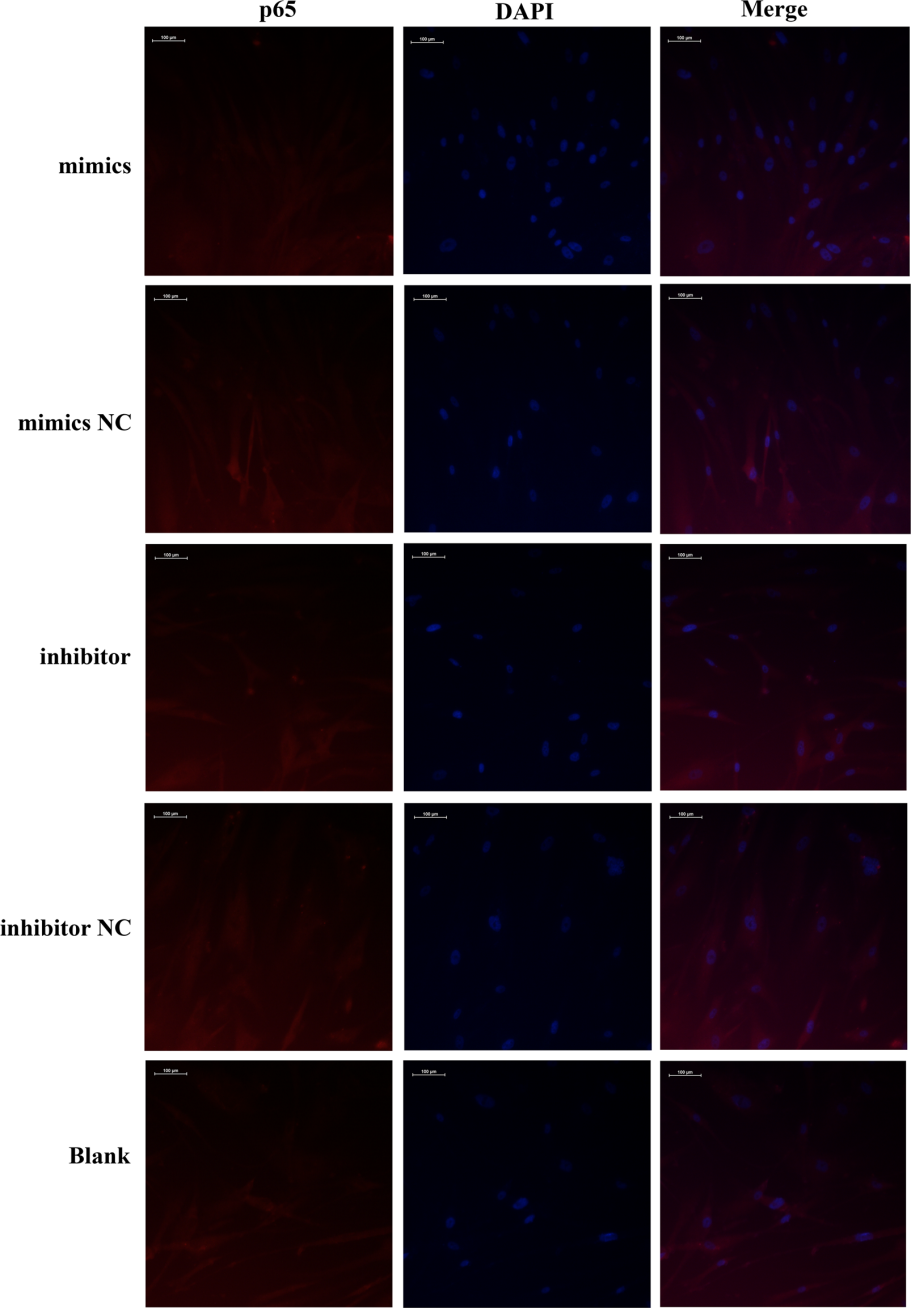
Effect of miR-410-3p on NF-κB activation. HFLS-RA were seeded in a 6-well culture plate, and subsequently treated with miR-410-3p mimics, inhibitor, or NC for 72 h. After treatment, cells were incubated with anti-p65 NF-κB antibody and Cy3 fluorescein-conjugated secondary antibody, and nuclei were stained with DAPI. The images were obtained by confocal laser microscopy and overlay. The pink fluorescence indicates location of p65 protein in nuclei.

To explore the potential mechanism underlying miR-410-3p-suppressed cytokine release in HFLS-RA, we conducted western blotting to test whether miR-410-3p regulates the NF-κB signaling pathway. The results showed that compared to the NC and blank groups, p-IκBα and p-p65 levels were significantly increased in the HFLS-RA transfected with miR-410-3p inhibitor (all *p* < 0.05) (Fig. 3a, b, d). In contrast, the total IκB-α and p65 levels showed no difference among the five groups (Fig. 3c, e). Furthermore, a nuclear translocation assay was performed to confirm the activation of NF-κB. The results showed that miR-410-3p upregulation suppressed the activation of NF-κB in HFLS-RA, while miR-410-3p inhibition activated NF-κB signaling (Fig. 4). These results indicate that in HFLS-RA, miR-410-3p overexpression suppressed the NF-κB signaling pathway.

### miR-410-3p Suppresses Cytokine Release in HFLS-RA by the NF-κB Signaling Pathway

**Fig. 5:**
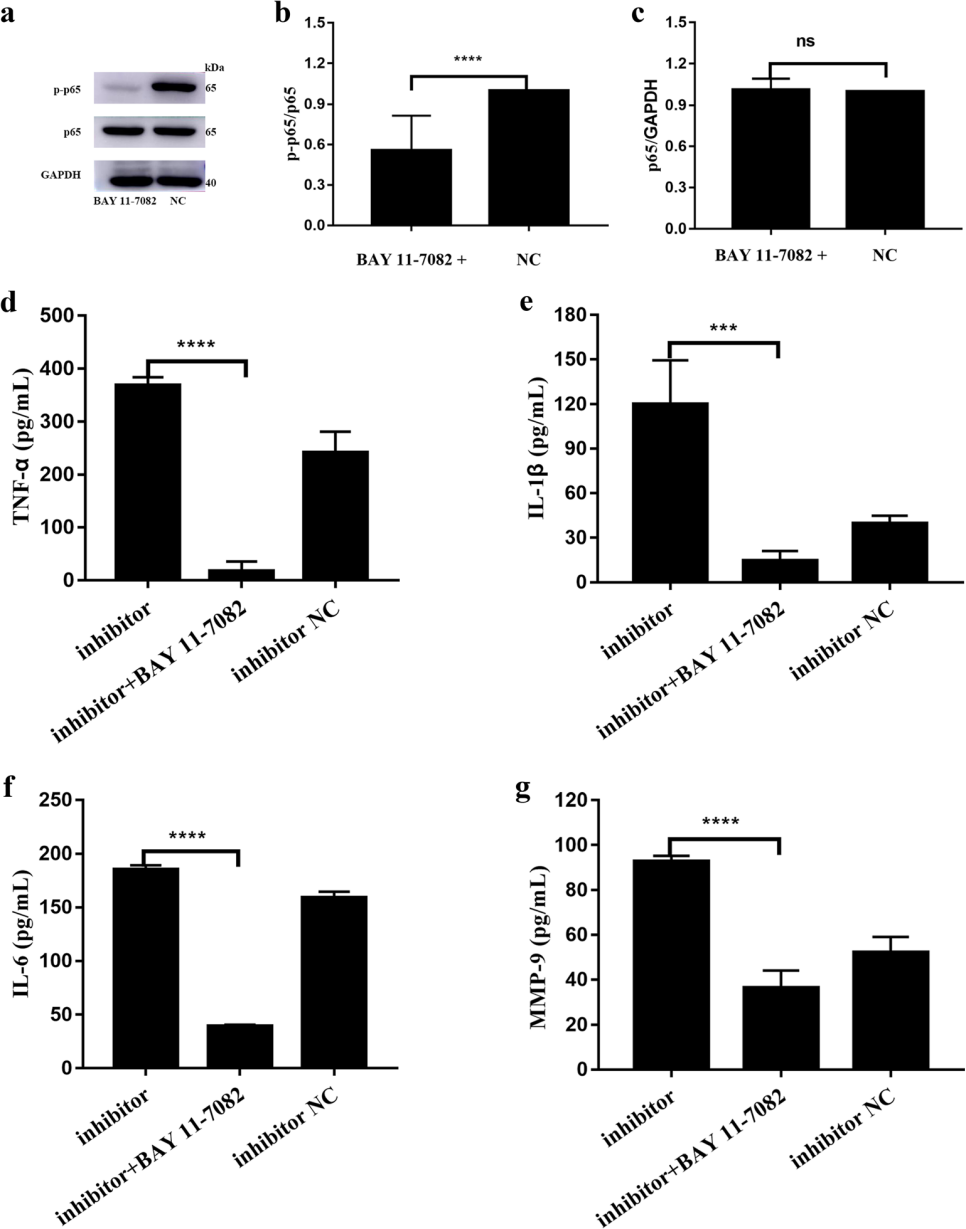
miR-410-3p suppresses cytokine release in HFLS-RA by the NF-κB signaling pathway. miR-410-3p inhibitor was transfected to HFLS-RA, with or without BAY 11-7082 treatment, followed by measuring the secretion of TNF-α, IL-1β, IL-6, and MMP-9 by ELISA. **a** Western blot of p-p65 and p65 after BAY 11-7082 treatment. BAY 11-7082 was dissolved in DMSO with the final concentration of 5 μM. Equal amount of DMSO was treated as the control group. **b**, **c** Histograms showing the expression of p-p65 and p65 levels detected by western blot. **d**–**g** Relative expression levels of TNF-α, IL-1β, IL-6, and MMP-9 in HFLS-RA after treatment. ***p < 0.001, ****p < 0.0001, compared with control.

Given the effect of miR-410-3p on the NF-κB signaling pathway, we further explored whether miR-410-3p suppressed cytokine release in HFLS-RA by regulating the NF-κB signaling pathway. To evaluate the involvement of NF-κB mediated by miR-410-3p, we pretreated the HFLS-RA with BAY 11-7082, an inhibitor of NF-κB nuclear translocation. As indicated in Fig. 5a–c, in the presence of BAY 11-7082, the levels of the p-p65 was significantly reduced (*p* < 0.0001), while total p65 levels showed no difference. Additionally, as shown in Fig. 5d–g, elevations of inflammatory cytokines, including TNF-α, IL-1β, IL-6, and MMP-9, in HFLS-RA induced by miR-410-3p inhibition was rescued by BAY 11-7082 (all *p* < 0.001), indicating that miR-410-3p suppressed cytokine release in HFLS-RA by regulating the NF-κB signaling pathway.

## DISCUSSION

RA is a systemic chronic inflammatory disease mainly characterized by hyperplastic synovial pannus tissue, which mediates the destruction of cartilage and bone [19]. FLSs are key components of this invasive synovium and play a major role in the initiation and perpetuation of destructive joint inflammation. As primary promoters of inflammation, FLSs from RA patients also display unique aggressive features. These FLS cells contribute to the inflammatory microenvironment and recruit and activate immune cells to the damaged synovium by secreting multiple pro-inflammatory cytokines and chemokines, particularly TNF-α, IL-1β, IL-6, and MMPs [20]. Elevated levels of cytokines and chemokines in the synovium modulate the expression of growth factors and result in further activation of FLSs, which undergo hyperplasia, a hallmark event in RA. Activated FLSs can undergo migration and enhance the production of pro-inflammatory cytokines and chemokines, particularly MMPs, which in turn mediate tissue destruction [21].

An increasing number of studies have focused on the abnormal expression of microRNAs in RA. Thus, understanding the disease-associated mechanisms of these small non-coding RNAs may provide a novel approach for the diagnosis and treatment of RA. Here, we showed that the expression of miR-410-3p was significantly lower in synovial tissues and FLSs of RA patients compared to that in healthy controls. Considering the downregulation of miR-410-3p in RA patients, this miRNA may have a critical role in RA development. Furthermore, we observed that miR-410-3p suppressed the inflammatory cytokine release of RA-FLS. Since abnormally activated NF-κB signaling pathway was responsible for the inflammation and excess proliferation of FLSs in RA [22], we further investigated whether miR-410-3p suppresses the cytokine release of RA-FLS by regulating the NF-κB signaling pathway.

miR-410-3p is abnormally expressed in a variety of diseases, including cancer, inflammation, and autoimmune diseases and is involved in multiple biological processes, such as proliferation, apoptosis, differentiation of stem cells, and drug resistance [13, 16, 23–25]. Previous studies showed that miR-410-3p was highly expressed in the lung and colorectal cancer, and promoted the proliferation, invasion, and migration of cancer cells [23, 26–28]. In contrast, miR-410-3p was reported to suppress the proliferation, migration, and invasion of breast cancer and pancreatic cancer [25, 29, 30]. These data indicate that dysregulation of miR-410-3p occurs in a tissue-specific manner in different types of diseases. A recent study showed that miR-410-3p attenuated renal fibrosis in lupus nephritis mice *via* directly targeting IL-6 [13]. Additionally, miR-410-3p was found to be involved in the development of osteoarthritis through modulating the differentiation of mesenchymal stem cells (MSCs) into chondrocytes [31]. However, the roles of miR-410-3p in RA are unknown. Nuclear translocation and subsequent DNA binding of NF-κB in inflamed synovium transactivate the expression of its target genes, including TNF-α, IL-1β and IL-6, and MMP-9 [32]. Therefore, NF-κB signaling has been extensively studied to identify its role in the pathogenesis of RA, and NF-κB inhibition has been examined as a therapeutic approach for the disease. Our results demonstrated that miR-410-3p suppressed NF-κB activation in cultured HFLS-RA by suppressing the phosphorylation of IκB-α, thus resulting in low phosphorylation of p65. As transcription targets, elevations of TNF-α, IL-1β, IL-6, and MMP-9 induced by miR-410-3p inhibition were restored by NF-κB inhibitor. Together, these findings suggest that miR-410-3p is involved in cytokine release by suppressing the NF-κB signaling pathway. These results suggest that miR-410-3p is an inflammatory suppressor in RA progression. However, there are still some limitations. First, the number of synovial tissue samples is relatively small. Additionally, certain animals’ experiments are needed to evaluate the effect of miR-410-3p on cytokine release *in vivo*.

Although miR-410-3p regulates multiple biological processes in a variety of diseases, its upstream regulating mechanism is unknown. It was reported that internalized miR-410-5p, derived from pre-miR-410, degraded miR-410-3p by base pairing and thus inhibited its function; blockade of the miR-410-5p upregulated the expression of miR-410-3p [14]. In contrast, CpG islands in the upstream regions of miR-410-3p were methylated; however, the expression level of miR-410-3p was not regulated by a DNA methylation inhibitor [33]. Additionally, some transcription factors were found to regulate the expression of miR-410-3p, such as myocyte enhancer factor 2 (MEF2) [34] and E2F [35]. However, little is known about the underlying mechanism of aberrant expression of miR-410-3p in RA, which requires further investigation.

In conclusion, we demonstrated that miR-410-3p acts as an inflammatory suppressor in RA by suppressing the NF-κB signaling pathway. These data suggest the potential diagnostic and therapeutic applications of miR-410-3p in RA.
